# Book Reviews

**Published:** 1868-10

**Authors:** 


### BIBLIOGRAPHICAL NOTICES.

*Dental Materia Medica.*—Compiled by James W. White, Published by S. S. White, Philadelphia. The receipt of many letters asking for information as to the properties and dental uses of many medicinal agents advertised for sale, induced the author to prepare this work, which will be found truly serviceable to every dental practitioner.

The author does not claim original ideas, but simply the collection from reliable authorities of a list of remedies in frequent use by the profession. Great credit is certainly due for the manner in which this work has been arranged, and also for the neat style in which it is gotten up.

The dental practitioner will find it an invaluable work for reference; and we understand that its author intends from time to time to issue new editions and thus keep up with the discoveries of the age. It contains one hundred and eight pages, its contents being Definitions of Terms, Abbreviations and Symbols, Weights and Measures, The Pulse and Respiration at various Ages, Eruption of the Teeth, and Dental Materia Medica.

*Physicians Visiting List for* 1869.—Lindsay & Blakiston, Philadelphia. This is one of the most complete Lists published, embracing an Almanac, Table of Signs, Hall's Ready Method in Asphyxia, Poisons and their Antidotes, Table for calculating the period of Utero-Gestation, Visiting List for 25 patients per week, and Blank Leaves for all the wants of the physician. It is printed on excellent paper and of a size and form well adapted to the purpose for which it is to be used.

We have also received from the same publishers their Annual Catalogue of medical works.

*I llustrated Catalogue of Med. Surg. and Scientific Publications*—Henry C. Lea, Philadelphia. This Catalogue is published in a neat form and contains a large number of illustrations taken from the works of this Publisner.

### EDITORIAL DEPARTMENT.

